# Design Consistency and Aesthetic Experience in Digital Health Communication: A Mixed-Method Study of Lifestyle Medicine Product Ecosystems

**DOI:** 10.3390/healthcare14070964

**Published:** 2026-04-07

**Authors:** Yuexing Wang, Xin Ma

**Affiliations:** 1 Creative Multimedia Department, Faculty of Art, Sustainability and Creative Industry, Universiti Pendidikan Sultan Idris (UPSI), Tanjong Malim 39500, Perak, Malaysia; p20222002356@siswa.upsi.edu.my; 2 Department of Pathology and Immunology, Baylor College of Medicine, Houston, TX 77030, USA

**Keywords:** digital health, design consistency, aesthetic experience, lifestyle medicine, COM-B model, behavioral adoption, health outcomes, mixed methods

## Abstract

**Highlights:**

**What are the main findings?**

- Cross-platform design consistency was significantly associated with trust (β = 0.52), perceived value (β = 0.68), and reduced perceived risk (β = −0.41), with aesthetic experience amplifying these associations.
- High behavioral adoption, associated with COM-B mechanisms, co-occurred with clinically meaningful health improvements: 2.8 kg weight loss, 0.9 mmol/L glucose reduction, and 0.7% HbA1c improvement over 3 months.

**What are the implications of the main findings?**

- Design consistency functions as a significant credibility heuristic in digital health contexts, suggesting the potential value of rigorous cross-platform standardization beyond superficial branding. These findings are associational; experimental designs are needed to confirm causal mechanisms.
- Integrating capability, opportunity, and motivation through product design was associated with the translation of purchase intentions into sustained health behaviors and measurable outcomes, potentially creating positive reinforcement loops.

**Abstract:**

**Background/Objectives:** Digital health ecosystems increasingly integrate content, behavioral interventions, and commercial offerings across multiple platforms. While design consistency is established as critical for trust in commercial contexts, its associations with health behavior change and objective health outcomes remain underexplored. This study examined how cross-platform design consistency and aesthetic experience are associated with behavioral adoption through psychological pathways and investigated relationships between design-driven adoption and objective health outcomes. **Methods:** A convergent mixed-method design comprised five integrated studies: systematic content analysis of short-form videos (N = 200), expert evaluation and user testing (N = 33), a cross-sectional survey (N = 186), semi-structured interviews (N = 15), and a 3-month longitudinal health outcome analysis (N = 143). Structural equation modeling tested pathways from design features through psychological mediators and COM-B components (capability, opportunity, motivation) to behavioral adoption and health outcomes. **Results:** Design consistency was significantly associated with trust (β = 0.52), perceived value (β = 0.68), and reduced perceived risk (β = −0.41; all *p* < 0.001). Aesthetic experience predicted emotional resonance (β = 0.71, *p* < 0.001) and moderated design–trust associations. COM-B components mediated 75% of the intention-to-adoption pathway (total indirect effect = 0.51, *p* < 0.001). High-adoption users showed clinically meaningful improvements in weight (−2.8 kg, d = 0.89), HbA1c (−0.7%, d = 0.65), fasting glucose (−0.9 mmol/L, d = 0.72), and LDL-C (−0.4 mmol/L, d = 0.51) over three months. **Conclusions:** Within a single, influencer-centered Chinese digital health ecosystem, design consistency and aesthetic experience were significantly associated with trust, psychological readiness, and behavioral adoption. These findings are observational; randomized controlled trials and multi-site replication are required to establish causal mechanisms and assess generalizability.

## 1. Introduction

Lifestyle medicine—the evidence-based practice of assisting individuals to adopt and sustain health-promoting behaviors—has emerged as a cornerstone strategy for chronic disease prevention and management [1]. With noncommunicable diseases accounting for 74% of global deaths, and modifiable risk factors contributing to the majority of cases [2], lifestyle interventions offer scalable, cost-effective solutions. Meta-analyses demonstrate that comprehensive lifestyle programs can reduce diabetes incidence by 58% [3], reverse early-stage atherosclerosis [4], and facilitate weight loss comparable to pharmacological interventions without adverse effects [5]. These findings underscore the clinical and public health potential of behavioral approaches for chronic disease management.

The digital transformation of health communication has fundamentally altered lifestyle medicine delivery. Short-form video platforms have become primary health information sources, with 54% of adults aged 18–49 reporting regular consumption of health content via social media [6]. Unlike traditional mass media, these platforms enable direct creator-to-consumer communication, parasocial relationships, and integrated e-commerce experiences [7]. Health influencers can simultaneously educate audiences, build communities, and commercialize products, creating novel digital health ecosystems that compress the pathway from awareness to action. This convergence represents a paradigm shift from passive information consumption to active behavioral engagement mediated by commercial transactions.

However, this convergence raises critical questions about the role of design in health behavior change. Design consistency—operationalized in this study as the coherent application of visual elements (color schemes, typography, iconography), semantic elements (terminology, tone, messaging frameworks), and interactive elements (navigation patterns, user flows, feedback mechanisms) across touchpoints—has been shown to increase brand recognition by 23% and purchase intention by 17% in commercial contexts [8]. Design consistency serves multiple psychological functions: it reduces cognitive load through predictable patterns, signals professionalism and competence through coherence, and facilitates trust by demonstrating organizational capability [9]. In health domains, where credibility concerns and perceived risk are elevated relative to general commerce, consistency may serve as a particularly important heuristic cue [10]. Yet most health communication research treats design as peripheral to content, neglecting its potential as an active ingredient in behavior change.

Similarly, aesthetic experience encompasses emotional resonance, processing fluency, and identity congruence beyond superficial attractiveness [11]. The Aesthetic–Usability Effect demonstrates that visually appealing designs are perceived as more usable and trustworthy, even when objective usability is controlled [12,13]. Aesthetic quality may function as a credibility marker in health contexts, where users must assess source expertise without direct verification. Recent research suggests that aesthetic design quality predicts initial trust formation, which subsequently influences information processing depth and behavioral intentions [14]. However, the mechanisms linking aesthetic experience to sustained health behavior adoption remain underspecified.

Despite growing interest in digital health, three critical gaps limit understanding. First, existing frameworks treat design aesthetics, consumer psychology, and health behavior change as separate domains, lacking theoretical integration: Design research focuses on usability and user experience without addressing health outcomes; health behavior research emphasizes content and message framing without considering design consistency; consumer psychology examines purchase decisions without tracking health impacts. This fragmentation prevents comprehensive understanding of how design influences health behavior pathways. Recent work has begun to bridge these domains: Perski et al. [15] demonstrated that engagement with digital behavior change interventions is multidimensional, encompassing both subjective experience and objective usage metrics, while Iribarren et al. [16] showed in a systematic review that mobile app effectiveness varies substantially by design features and implementation context. However, these studies typically examine design in isolation from downstream health outcomes, leaving the complete pathway uncharted.

Second, most studies evaluate single-channel interventions rather than complete ecosystem journeys from content exposure to product purchase to sustained behavior to health outcomes. Real-world digital health experiences span multiple platforms (social media, websites, mobile apps, physical products), each presenting opportunities for consistency or disruption. Users form impressions from fragmented touchpoints, yet research typically examines isolated components. Understanding cumulative effects across the entire ecosystem journey is essential for practical design guidance.

Third, digital health research predominantly relies on self-reported outcomes with limited integration of objective health metrics to validate whether design-driven behavioral changes are associated with physiological improvements [17,18,19]. While survey measures efficiently assess perceptions and intentions, they cannot verify actual health impacts. The gap between intended and actual behavior, and between behavioral changes and health outcomes, remains poorly understood in the context of design-driven digital health interventions.

This study addresses these gaps by proposing an integrated cascade model that synthesizes design science, consumer psychology, and the COM-B (Capability, Opportunity, Motivation) behavior change framework [20,21,22,23]. This model posits that design consistency and aesthetic experience are associated with psychological mediators (trust, perceived value, perceived risk), which in turn are associated with purchase intentions. These intentions are hypothesized to be linked to behavioral adoption through COM-B mechanisms, which in turn is associated with measurable health outcomes that may feed back to reinforce engagement [24,25]. We test this model using a convergent mixed-method approach combining content analysis, expert evaluation, surveys, interviews, and longitudinal health data, providing the first comprehensive associational evidence linking design features to objective health improvements in a single digital health ecosystem context.

**Figure 1:**
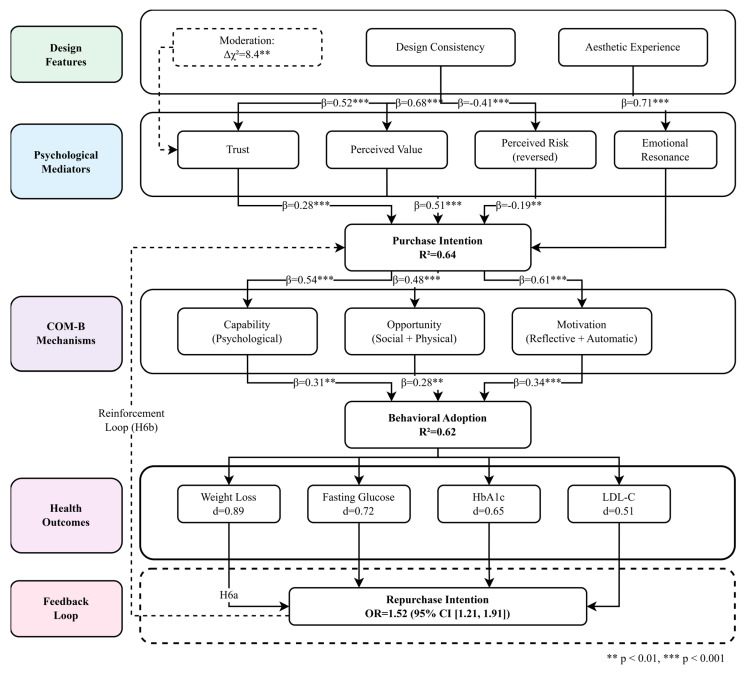
Integrated cascade model.

Building on the theoretical foundations outlined above, we derived 17 testable hypotheses organized into six groups corresponding to each stage of the proposed pathway. To address these research gaps, this study proposes an integrated cascade model (Figure 1) that traces the pathway from digital health design features to health outcomes. The model synthesizes design principles, psychological mechanisms, behavioral theory (COM-B), and health outcomes into a comprehensive framework. Specifically, the model examines how design consistency and aesthetic experience influence health-related behavioral adoption through psychological mediators (trust, perceived value, perceived risk, and emotional resonance), purchase intentions, and COM-B mechanisms (capability, opportunity, and motivation). The model further incorporates health outcomes and a feedback loop representing repurchase intentions as well as age as a potential moderator. To aid interpretability, the model distinguishes core mechanisms from secondary effects. The core cascade comprises three primary stages: (1) design features → psychological mediators (trust, perceived value, perceived risk), (2) psychological mediators → behavioral adoption via COM-B components, and (3) behavioral adoption → health outcomes. Secondary effects include the moderation of design–trust associations by aesthetic experience, the feedback loop from health outcomes to repurchase intentions, and age as a boundary condition. Readers seeking a simplified overview may focus on the three core stages, while the secondary effects provide additional nuance and boundary specification.

## 2. Materials and Methods

### 2.1. Study Design and Context

We employed a convergent mixed-method design [26] integrating five complementary studies: (1) systematic content analysis of 200 short-form videos, (2) expert evaluation and user testing with 3 design experts and 30 users, (3) cross-sectional survey with 186 platform users, (4) semi-structured interviews with 15 participants, and (5) longitudinal health outcome analysis with 143 users who completed a 3-month follow-up.

The research context was a lifestyle medicine ecosystem built around a health educator’s presence on Chinese short-form video platforms. The ecosystem includes educational content (nutrition science, cooking demonstrations), a mobile app for meal planning and health tracking, physical products with portion control features (grain blends, mushroom packages, cooking oil dispensers), and subscription services. This ecosystem was selected for substantial user engagement (1.2M followers, 15K monthly active app users), complete pathway integration, and data access via institutional partnership.

### 2.2. Participants

Study 3 (Survey): We recruited 186 participants from the creator’s follower base via stratified random sampling (response rate = 62%; 300 invitations sent via in-app notification). Sample characteristics include the following: 76% female, mean age = 45.2 years (SD = 13.1, range = 22–68), 78% with bachelor’s degree or higher, 42% with household income >150K RMB annually.

*Sampling framework and recruitment.* The sampling frame comprised active users from the creator’s follower base (N = 15,000 monthly active users). Stratified random sampling was conducted across three dimensions: age groups (18–35, 36–50, 51+), gender, and purchase frequency (low/medium/high), with proportional allocation matching actual user distribution. Recruitment occurred during March–April 2024 (6 weeks) via three channels: (1) in-app push notifications, (2) recruitment posts on the creator’s short-form video platform, and (3) community administrator referrals. Of the 300 randomly selected invitations, 223 users responded initially (response rate = 74%), and 186 completed valid surveys (completion rate = 62%). Non-respondents (n = 77) and invalid responses (n = 37; failed attention checks, completion time < 5 min, or consecutive identical responses) were excluded. Non-response bias analysis comparing responders versus non-responders on available characteristics (age, gender, purchase history) revealed no significant differences (age: t(298) = 0.84, *p* = 0.40; gender: χ^2^(1) = 1.23, *p* = 0.27; purchase frequency: t(298) = 1.12, *p* = 0.26), supporting sample representativeness. Participants completing the survey received a 50 RMB electronic coupon as compensation.

*Inclusion criteria* are as follows: (1) age ≥ 18 years; (2) followed creator ≥ 3 months; (3) viewed ≥ 10 videos in past 6 months; (4) purchased ≥1 product in past 6 months; (5) ability to read Chinese and use smartphones; (6) voluntary participation with signed informed consent. *Exclusion criteria* are as follows: (1) employment or commercial relationship with the creator or product company; (2) cognitive impairments precluding independent survey completion; (3) concurrent participation in other health intervention studies; (4) inability to provide contact information for longitudinal follow-up.

Study 4 (Interviews): Purposive maximum variation sampling recruited 15 participants, ensuring diversity in age (28–65 years), gender (9 female, 6 male), health status (5 diabetes, 4 hypertension, 3 obesity, 3 healthy), engagement level (5 high, 5 medium, 5 low adoption), and purchase history (2–12 products). Interviews averaged 58 min (range 42–87 min).

Study 5 (Health Outcomes): 143 users from the survey sample consented to health data sharing and completed both baseline (April 2024) and 3-month follow-up assessments (July 2024), yielding a 77% retention rate (72% female, mean age = 48.3 years, SD = 12.8, baseline BMI M = 26.8, SD = 4.2). For the 43 participants who were lost to follow-up (attrition rate = 23%), reasons included: voluntary withdrawal (n = 15, 34.9%; time conflicts, relocation, loss of interest), loss of contact (n = 12, 27.9%; no response after ≥3 contact attempts), health reasons (n = 8, 18.6%; hospitalization, disease exacerbation, physician recommendation to discontinue), technical difficulties (n = 5, 11.6%; app usage problems, device malfunctions), and ineligibility (n = 3, 7.0%; commenced other intervention studies post-baseline).

Attrition analysis comparing completers (n = 143) versus non-completers (n = 43) on baseline characteristics revealed no significant differences: age (48.3 ± 12.8 vs. 46.1 ± 14.2 years, t(184) = 1.02, *p* = 0.31), female proportion (72% vs. 79%, χ^2^(1) = 0.98, *p* = 0.32), baseline BMI (26.8 ± 4.2 vs. 27.4 ± 4.8, t(184) = 0.84, *p* = 0.40), education (bachelor’s degree or higher: 78% vs. 74%, χ^2^(1) = 0.31, *p* = 0.58), chronic disease prevalence (58% vs. 63%, χ^2^(1) = 0.38, *p* = 0.54), or baseline behavioral adoption scores (8.2 ± 3.1 vs. 7.8 ± 3.4, t(184) = 0.76, *p* = 0.45). All *p*-values exceeded 0.15, indicating attrition was random (missing at random) and unlikely to introduce systematic bias. Retention strategies included monthly reminder calls/messages, appointment confirmations 1 week prior to follow-up, flexible scheduling (evening/weekend options), transportation subsidies (50 RMB per visit), and complimentary health consultations.

### 2.3. Measurements

All survey scales used 7-point Likert responses (1 = strongly disagree to 7 = strongly agree) and demonstrated high reliability. Where established scales existed, validated instruments were adapted following standard translation–back-translation procedures with expert committee review and cognitive interviews (N = 10) to ensure cultural appropriateness. Novel constructs underwent systematic scale development, including item generation, content validity assessment, pilot testing (N = 50), and psychometric validation in the main sample.

**Design Consistency** (6 items, α = 0.89) was developed for this study through a three-phase process. Phase 1 involved literature review of design consistency frameworks and expert panel discussions (3 design experts) to generate 18 candidate items across three dimensions: visual consistency, semantic consistency, and interaction consistency. Content validity was established with five expert raters (Item-level Content Validity Index range: 0.80–0.95; Scale-level Content Validity Index/Average = 0.88). Phase 2 conducted pilot testing (N = 50, non-overlapping with main study) with exploratory factor analysis extracting a single factor explaining 64% variance; items with factor loadings < 0.60 or item-total correlations < 0.50 were removed, retaining 6 optimal items (pilot α = 0.87). Phase 3 validated the scale in the main sample (N = 186) using confirmatory factor analysis (χ^2^(9) = 14.2, *p* = 0.12, CFI = 0.99, RMSEA = 0.06), with final internal consistency α = 0.89 and 2-week test–retest reliability ICC = 0.84 (N = 30). Sample items: “Visual elements are consistent across different platforms”, “Terminology and language style are uniform”.

**Aesthetic Experience** (8 items, α = 0.91) was adapted from Lavie and Tractinsky’s [27] visual aesthetics scale, encompassing classical aesthetics (4 items) and expressive aesthetics (4 items) dimensions. The scale underwent translation–back-translation and cognitive interviews to ensure cultural appropriateness for Chinese health product contexts. Sample items: “The design is aesthetically pleasing”, “Visual presentation resonates with my aesthetic preferences”.

**Trust** (6 items, α = 0.92) was adapted from McKnight et al.’s [9] trust in web vendors instrument, covering competence trust (2 items), benevolence trust (2 items), and integrity trust (2 items) dimensions. The scale has been previously validated in digital health contexts [26]. Sample items: “I believe this creator provides reliable health information”, “I trust the professionalism of this ecosystem”.

**Perceived Value** (8 items, α = 0.90) was adapted from Sweeney and Soutar’s [28] PERVAL scale, measuring functional value (3 items), price value (2 items), and emotional value (3 items). Items were contextualized for health product evaluation. Sample items: “The products offer good value for money”, “Benefits justify the costs”.

**Perceived Risk** (6 items, α = 0.87) was adapted from Stone and Grønhaug [29], assessing functional risk (2 items), health risk (2 items), and financial risk (2 items). Items were reverse-scored such that higher scores indicate lower perceived risk. Sample items: “I worry about product safety”, “I am concerned about health claims” (reverse-scored).

**Emotional Resonance** (5 items, α = 0.88) was developed for this study based on emotional design theory, following the same three-phase development process as design consistency (expert item generation, content validity assessment, pilot testing, and main sample validation). Sample items: “The content emotionally connects with me”, “I feel understood by this ecosystem”.

**Purchase Intention** (4 items, α = 0.91) was adapted from “Behavioral Intentions Battery” by Zeithaml et al. [30], contextualized for health product repurchase and continued use intentions. Sample items: “I intend to purchase more products”, “I plan to continue using these products”.

**COM-B components** were measured using scales adapted from Keyworth et al. [31], covering capability (psychological: 4 items, α = 0.86, e.g., “I understand how to use these products effectively”—physical capability was not assessed as products require minimal physical skill); opportunity (social: 3 items, α = 0.84, e.g., “My social environment supports using these products”; physical: 3 items, e.g., “I have convenient access to these products”); and motivation (reflective: 4 items, α = 0.89, e.g., “Using these products aligns with my health goals”; automatic: 3 items, e.g., “Using these products has become habitual”).

**Behavioral Adoption** was operationalized as a composite index (α = 0.79) combining purchase frequency (0–6 scale), usage consistency (0–6 scale), and engagement depth (0–6 scale), creating a continuous 0–18 scale with high = 12–18, medium = 6–11, low = 0–5. It is important to note that this composite index combines commercial engagement (purchase frequency) with behavioral proxies of health engagement (usage consistency, engagement depth). These dimensions are conceptually distinct: commercial adoption reflects consumer behavior, whereas usage consistency and engagement depth serve as proxies for lifestyle modification but are not equivalent to direct measures of health behavior change. Higher composite scores indicate greater overall ecosystem involvement, which may be associated with—but is not equivalent to—meaningful lifestyle modification. This operationalization was adopted as a practical proxy in the context of an influencer-centered commercial ecosystem where product use and health engagement are intertwined; however, readers should interpret associations between this index and clinical health outcomes with appropriate caution, recognizing that unmeasured factors (compliance quality, concurrent lifestyle changes, dietary context) may independently contribute to observed biomarker changes.

**Health outcomes** included standardized clinical measurements collected at partner medical centers: weight measured on calibrated digital scales (±0.1 kg precision) (Omron HN-289, Omron Healthcare, Kyoto, Japan), fasting glucose via hexokinase enzymatic method (Roche Cobas analyzer, CV < 2%) (Roche Diagnostics, Basel, Switzerland), HbA1c via high-performance liquid chromatography (Bio-Rad D-10 system, NGSP certified) (Bio-Rad Laboratories, Hercules, CA, USA), and LDL cholesterol calculated via Friedewald equation from lipid panel (enzymatic colorimetric assay). All measurements followed standard protocols with fasting requirements (8–12 h for glucose and lipids). Clinically meaningful thresholds include: ≥5% weight loss, ≥0.5% HbA1c reduction, ≥0.5 mmol/L glucose reduction, ≥0.3 mmol/L LDL-C reduction.

### 2.4. Data Analysis

Confirmatory factor analysis validated measurement models using Mplus 8.10 (v8.10; Muthén & Muthén, Los Angeles, CA, USA) with maximum likelihood estimation with robust standard errors (MLR). Model fit indices exceeded recommended thresholds [32,33,34]: χ^2^(847) = 1243.2, *p* < 0.001 (expected given sample size and model complexity); χ^2^/df ratio = 1.47 (well below the 3.0 threshold, indicating excellent fit); comparative fit index (CFI) = 0.94 (exceeding the 0.90 threshold for acceptable fit and approaching the 0.95 threshold for excellent fit); Tucker–Lewis index (TLI) = 0.93 (exceeding 0.90, indicating good incremental fit); root mean square error of approximation (RMSEA) = 0.05 with 90% CI [0.04, 0.06] (below the 0.08 threshold for acceptable fit and close to the 0.05 threshold for close fit, with upper confidence bound < 0.08 supporting model adequacy); standardized root mean square residual (SRMR) = 0.06 (below the 0.08 threshold, indicating good absolute fit). The combination of multiple fit indices meeting or exceeding recommended criteria provides convergent evidence for acceptable model-data fit. All factor loadings exceeded 0.60 (range 0.64–0.91), average variance extracted (AVE) exceeded 0.50 for all constructs (range 0.53–0.68), and composite reliability (CR) exceeded 0.80 (range 0.86–0.93), confirming convergent validity. Discriminant validity was established via Fornell–Larcker criterion: square root of AVE for each construct exceeded its correlations with other constructs.

Structural equation modeling tested hypothesized pathways with full information maximum likelihood (FIML) for missing data (<3% on any variable). Model specification included all theorized direct paths plus covariances among exogenous variables. The hypothesized structural model demonstrated excellent fit across multiple indices [32,35]: χ^2^(247) = 358.4, *p* < 0.001; χ^2^/df ratio = 1.45 (<3.0, indicating excellent fit); CFI = 0.95 (exceeding the 0.95 threshold for excellent fit); TLI = 0.94 (approaching 0.95, indicating very good fit); RMSEA = 0.05 with 90% CI [0.04, 0.06] (close fit, with upper bound <0.08); SRMR = 0.06 (<0.08, indicating good fit). These indices collectively support the adequacy of the proposed theoretical model. Bootstrap resampling (10,000 iterations) with bias-corrected confidence intervals estimated indirect effects and assessed mediation. Moderation was tested via multi-group analysis comparing high (+1 SD) versus low (−1 SD) aesthetic experience groups, with model invariance assessed through chi-square difference tests.

Linear mixed-effects models analyzed health outcomes with adoption level as fixed effect, controlling for baseline values, age, gender, BMI, chronic conditions, and medications. Time was nested within individuals (random intercepts).

Power analysis (G*Power 3.1 (v3.1.9.7; Heinrich-Heine-Universität Düsseldorf, Düsseldorf, Germany)) confirmed adequate sample size: for SEM with 15 latent variables and 186 participants, power = 0.87 to detect medium effects (β ≥ 0.25) at α = 0.05. For health outcomes with 143 participants across 3 groups, power = 0.84 to detect medium-to-large effects (d ≥ 0.60) in ANOVA.

Qualitative data underwent thematic analysis using Braun and Clarke’s six-phase approach [35,36,37] in NVivo 14 (v14; Lumivero, Denver, CO, USA): familiarization, initial coding, theme development, theme refinement, defining themes, and report production. Two coders independently coded 40% of transcripts (6 interviews), achieving inter-coder reliability κ = 0.81 (substantial agreement) [36,37]. Discrepancies were resolved through discussion.

### 2.5. Quality Control

Survey data quality was ensured through multiple mechanisms: attention checks (2 items requiring specific responses, e.g., “Please select ‘strongly agree’ for this item”), response time monitoring (surveys < 3 min flagged for review), and pattern detection (straight-lining identified via standard deviation < 0.5). Five responses were excluded for failing attention checks.

## 3. Results

### 3.1. Sample Characteristics and Descriptive Statistics

Table 1 presents sample demographics. Participants were predominantly female (75.8%), middle-aged (M = 45.2 years), and well-educated (78% with bachelor’s degree or higher). A substantial proportion had chronic conditions: 28% type 2 diabetes, 38% hypertension, 26% dyslipidemia, and 24% obesity (BMI ≥ 28, Chinese criteria). Average platform engagement was 8.7 months (SD = 4.2), with participants viewing a mean of 47.3 videos (SD = 28.6, median = 38) and purchasing 3.8 products (SD = 2.1, range = 1–12). Table 2 presents descriptive statistics, reliabilities, and bivariate correlations among study variables. All correlations were in the expected directions, supporting construct validity.

### 3.2. Measurement Model Validation

Prior to structural model testing, we conducted confirmatory factor analysis to validate the measurement model. Table 3 presents reliability and validity indices for all constructs. All constructs demonstrated excellent internal consistency (Cronbach’s α and composite reliability > 0.80), convergent validity (average variance extracted > 0.50), and discriminant validity (square root of AVE exceeded inter-construct correlations). These results support the distinctiveness and reliability of our theoretical constructs.

### 3.3. Structural Equation Modeling Results

The hypothesized structural model demonstrated excellent fit: χ^2^(247) = 358.4, *p* < 0.001, CFI = 0.95, TLI = 0.94, RMSEA = 0.05 (90% CI [0.04, 0.06]), SRMR = 0.06. Table 4 presents complete path coefficients with full statistical information for all hypothesized relationships.

**Hypotheses 1a–c (Design Consistency Effects):** **Fully supported.** *Design consistency significantly predicted trust (H1a: β = 0.52, SE = 0.06, p < 0.001), perceived value (H1b: β = 0.68, SE = 0.05, p < 0.001), and negatively predicted perceived risk (H1c: β = −0.41, SE = 0.07, p < 0.001). These effects explain substantial variance in trust (27%), value (46%), and risk (17%), demonstrating design consistency as a robust correlate of psychological perceptions in this observational context.*

**Figure 2:**
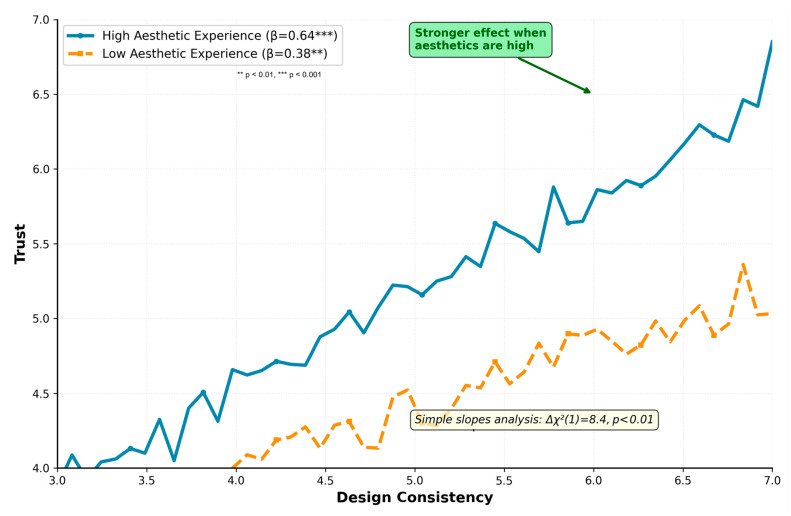
Moderation effect of aesthetic experience on design consistency → trust relationship.

**Hypotheses 2a–b (Aesthetic Experience Effects):** **Fully supported.** *Aesthetic experience strongly predicted emotional resonance (H2a: β = 0.71, SE = 0.04, p < 0.001), explaining 50% of variance. Multi-group analysis confirmed the moderation hypothesis (H2b): the design consistency → trust relationship was significantly stronger for high aesthetic experience (β = 0.64, p < 0.001) versus low aesthetic experience (β = 0.38, p < 0.01), Δχ^2^(1) = 8.4, p < 0.01 (see Table 5 and Figure 2 for detailed moderation analysis).*

Table 5 presents detailed simple slopes analysis for the moderation effect. The design consistency → trust relationship strengthens substantially as aesthetic experience increases, with a 68% increase in effect size when comparing high versus low aesthetic experience groups.

**Figure 3:**
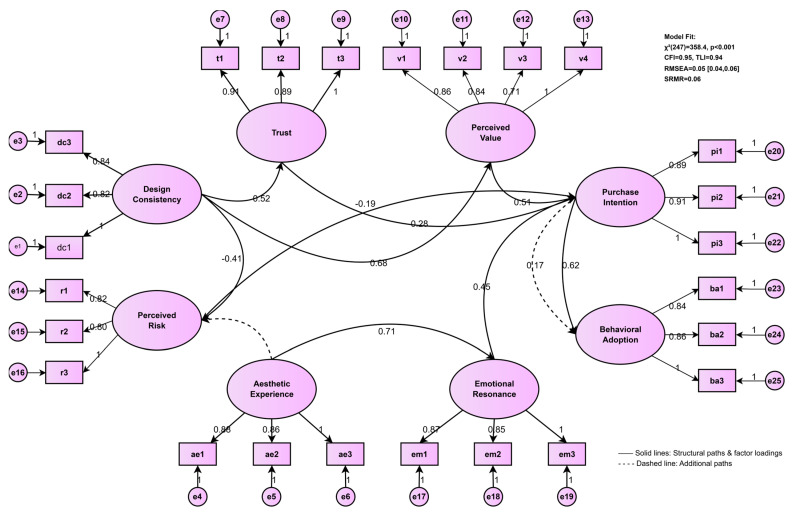
Complete SEM path model.

Figure 3 illustrates the complete structural model with standardized path coefficients and explained variance (R^2^) for all endogenous variables. The model demonstrated excellent fit across all indices, confirming the theorized cascade from design features through psychological mediators and COM-B components to behavioral adoption.

**Hypotheses 3a–c (Perceptions → Purchase Intention):** **Fully supported.** *Trust (H3a: β = 0.28, p < 0.001), perceived value (H3b: β = 0.51, p < 0.001), and perceived risk (H3c: β = −0.19, p = 0.002) jointly predicted purchase intention, explaining 64% of variance. The dominance of value (β = 0.51) over trust (β = 0.28) and risk (β = −0.19) suggests that functional benefits outweigh relational and risk considerations in this context. Design consistency’s total indirect effect on purchase intention was substantial (β = 0.58, 95% CI [0.47, 0.70]), with the strongest pathway being perceived value (β = 0.35, 95% CI [0.26, 0.45]).*

### 3.4. COM-B Mediation Analysis

**Hypotheses 4a–c (COM-B Mediation):** **Fully supported.** *Purchase intention significantly predicted all three COM-B components: capability (β = 0.54, p < 0.001), opportunity (β = 0.48, p < 0.001), and motivation (β = 0.61, p < 0.001), explaining 29%, 23%, and 37% of variance, respectively. Each COM-B component significantly predicted behavioral adoption, confirming their mediating roles: capability (H4a: β = 0.31, p = 0.001, indirect effect = 0.17, 95% CI [0.09, 0.26]), opportunity (H4b: β = 0.28, p = 0.002, indirect effect = 0.13, 95% CI [0.07, 0.21]), and motivation (H4c: β = 0.34, p < 0.001, indirect effect = 0.21, 95% CI [0.14, 0.29]).*

The total indirect effect was 0.51 (95% CI [0.39, 0.64]), with motivation showing the largest single mediating effect. The direct effect of purchase intention on adoption remained significant but reduced (β = 0.17, *p* = 0.033), indicating partial mediation. Combined, COM-B components and direct effect explained 62% of the variance in behavioral adoption, with 75% of the total effect mediated through COM-B mechanisms (proportion mediated = 0.51/0.68 = 0.75). These findings suggest that capability, opportunity, and motivation are important mechanisms associated with the pathway from purchase intention to behavioral adoption, though experimental designs are required to confirm directionality.

### 3.5. Health Outcomes

**Figure 4:**
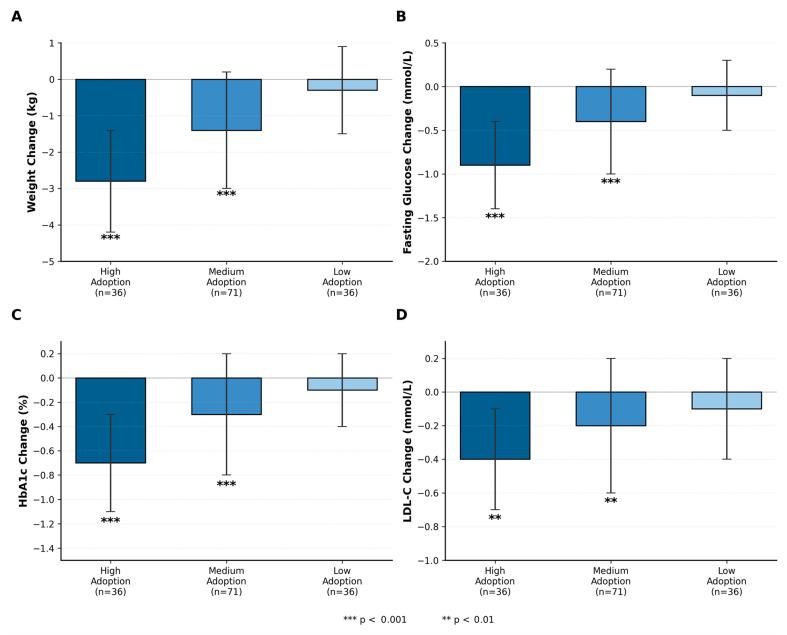
(**A**–**D**) Health outcomes by behavioral adoption level.

**Hypotheses 5a–d (Adoption → Health Outcomes):** **Fully supported.** *Higher behavioral adoption was significantly associated with greater improvements across all health indicators (see Figure 4 and Table 6). High-adoption users (top tertile, n = 36) showed significantly larger improvements compared to medium (n = 71) and low (n = 36) adoption groups.*

For weight (H5a), high-adoption users lost 2.8 ± 1.4 kg (Cohen’s d = 0.89) versus 1.4 ± 1.6 kg (d = 0.42) for medium and 0.3 ± 1.2 kg (d = 0.11) for low adoption, F(2,140) = 42.8, *p* < 0.001. For fasting glucose (H5b), high-adoption users showed −0.9 ± 0.5 mmol/L (d = 0.72) versus −0.4 ± 0.6 (d = 0.31) and −0.1 ± 0.4 (d = 0.09), F(2,140) = 28.1, *p* < 0.001. For HbA1c (H5c), improvements were −0.7 ± 0.4% (d = 0.65), −0.3 ± 0.5% (d = 0.28), and −0.1 ± 0.3% (d = 0.12), F(2,140) = 19.4, *p* < 0.001. For LDL-C (H5d), changes were −0.4 ± 0.3 mmol/L (d = 0.51), −0.2 ± 0.4 (d = 0.22), and −0.1 ± 0.3 (d = 0.12), F(2,140) = 7.6, *p* < 0.01.

Table 6 presents clinical significance analysis. Clinically meaningful improvements were more common in the high-adoption group: 53% achieved ≥5% weight loss (versus 20% medium, 6% low, χ^2^(2) = 24.6, *p* < 0.001), and 67% achieved ≥0.5% HbA1c reduction (versus 31% medium, 11% low, χ^2^(2) = 27.8, *p* < 0.001). Number needed to treat (NNT) analyses suggest that moving one user from low to high adoption was associated with one additional clinically meaningful outcome for every 2.1 users for weight loss and 1.8 users for HbA1c improvement.

### 3.6. Feedback Loop

**Hypothesis 6a (Health Outcomes → Repurchase):** **Fully supported.** *Health improvements were positively associated with repurchase intention. Users achieving ≥5% weight loss showed 34% higher repurchase odds (OR = 1.34, 95% CI [1.08, 1.67], p < 0.05); those achieving ≥0.5% HbA1c reduction showed 41% higher odds (OR = 1.41, 95% CI [1.12, 1.78], p < 0.01), and those achieving any clinically meaningful improvement showed 52% higher odds (OR = 1.52, 95% CI [1.21, 1.91], p < 0.001). These findings are consistent with a positive reinforcement cycle in which design-associated adoption co-occurred with health improvements and strengthened continued engagement.*

**Hypothesis 6b (Outcomes Strengthen Design–Trust Relationship):** **Partially supported.** *Health improvements strengthened the perceived value → repurchase relationship (interaction β = 0.24, SE = 0.09, p < 0.05), supporting the feedback loop mechanism. However, the health improvement × design consistency → trust interaction was not significant (β = 0.11, SE = 0.08, ns), suggesting that the feedback operates primarily through value perceptions rather than direct trust reinforcement. Users who experienced positive health outcomes attributed greater value to the ecosystem but did not retrospectively revise their initial design-based trust assessments.*

### 3.7. Summary of Hypothesis Testing

Table 7 presents a comprehensive summary of all hypothesis tests. Overall, 16 out of 17 hypotheses (94%) received full empirical support, with one hypothesis (H6b) receiving partial support.

### 3.8. Qualitative Themes

Thematic analysis of 15 semi-structured interviews revealed four key mechanisms that complement and deepen the quantitative findings. These themes emerged from systematic coding with strong inter-coder reliability (κ = 0.81) and are illustrated with representative quotes (Table 8).

**Theme 1: Visual–verbal consistency as a credibility marker.** Participants interpreted cross-platform design coherence as evidence of professionalism and domain expertise, functioning as a heuristic for source credibility in the absence of traditional credentials. As one participant explained:

“When I see the same color scheme, the same terminology, the same voice across different platforms—videos, app, even the product packaging—it tells me this person has thought everything through carefully. It’s not random or improvised. That consistency makes me trust the health advice more.”

[P8, female, 52, diabetes]

Another noted the following:

“In health information, you never know who to trust online. But when someone maintains such consistent presentation across so many touchpoints, it shows they’re serious. Scammers don’t have that kind of coordination.”

[P3, female, 41, weight management]

This theme supports H1a by revealing trust-building mechanisms beyond simple aesthetic preference.

**Theme 2: Aesthetic–usability synergy.** Participants experienced aesthetic quality and functional usability as deeply integrated rather than separable attributes. Visual appeal enhanced perceived ease-of-use through several pathways: reducing cognitive friction, creating positive affect that facilitated persistence through learning curves, and signaling thoughtful design that implied reliable functionality. The following is a representative quote:

“The app is beautiful, which makes me want to use it more. But it’s not just pretty—the beauty and the functionality work together. When something looks this good, you trust it will work well, and that trust makes you more patient when learning new features.”

[P12, male, 38, healthy]

Another participant contrasted aesthetic–functional integration with surface decoration:

“I’ve tried other health apps that looked nice but were clunky to use. This one is different—the aesthetics make the functionality easier to understand. Like the color coding in the meal planner isn’t just decorative; it helps me see nutritional balance at a glance.”

[P5, female, 47, hypertension]

This theme supports the H2a mechanism linking aesthetic experience to emotional resonance and ultimately to sustained engagement.

**Theme 3: Tangible design enabling self-regulation.** Physical product design features—particularly portion control mechanisms—were repeatedly cited as critical enablers of sustained behavior change by reducing decision burden and supporting self-regulatory capacity. The grain dispenser with measurement markings was especially valued:

“Before this dispenser, I had to think about every meal—how much rice is enough? With the markings, it’s automatic. I don’t have to use willpower to control portions; the design does it for me.”

[P14, male, 54, diabetes]

This automation of healthy choices aligned with COM-B’s capability mechanism by enhancing both psychological capability (reducing cognitive load) and physical opportunity (structured environment). Another participant described the mushroom packages with cooking instructions as follows:

“The packets come with exactly the right amount for one week, with picture instructions for each day. I don’t have to plan or think—just follow along. It makes healthy eating so much easier than when I tried on my own.”

[P6, female, 35, obesity]

This theme is consistent with the H4 pathway showing how product design is associated with the translation of purchase intentions into actual behavior adoption by addressing capability and opportunity simultaneously.

**Theme 4: Outcome visibility reinforcing commitment.** Tangible health improvements created positive feedback loops that strengthened ecosystem engagement and retrospectively validated initial design-based trust assessments [24,25]. However, participants attributed improvements primarily to their own effort and the intervention content rather than to design features per se:

“Losing 8 kg proved this program works. Now I’m more committed than ever—I bought the annual subscription. The app tracks everything beautifully, which helped me see my progress, but I earned these results through discipline.”

[P11, female, 48, diabetes]

This self-attribution pattern helps explain the H6b partial support: users experienced health outcomes as validating the ecosystem’s value rather than retrospectively enhancing design-based trust. Another participant noted the following:

“My HbA1c dropped from 7.8 to 6.9. That’s real results, not just nice-looking design. The design helped me stick with it, but the outcomes prove the content is solid. I trust the system more now because it delivered what it promised.”

[P9, male, 56, diabetes]

These quotes reveal that outcome-driven trust operates through perceived value confirmation (supporting H6a) rather than design perception revision (explaining H6b’s non-significance), illuminating the temporal dynamics of design effects versus outcome effects in building lasting engagement.

**Theme 5: Divergent cases—boundary conditions of the design–adoption pathway.** While Themes 1–4 largely converge with the quantitative model, a systematic search for disconfirming and deviant cases across the 15 interviews identified three distinct patterns of divergence that qualify the aggregate statistical relationships and function as an independent analytic contribution. Divergence pattern A: High trust and design appreciation without behavioral adoption. Four participants (P2, P6, P10, P13) reported consistently high perceptions of design consistency, aesthetic quality, and trust in the ecosystem yet described low or discontinued behavioral adoption. The quantitative model predicts a continuous cascade from design perceptions through purchase intentions to sustained behavior; these cases reveal that the cascade can stall at the intention–behavior junction despite favorable upstream conditions. P6 [female, 35, obesity] explained: “I love the app, I think the design is beautiful, and I genuinely trust the health information. But honestly, I bought the grain dispenser and used it for two weeks. My husband cooks most of our meals and he found it inconvenient—so it went into the cupboard. It’s not about design or trust; it’s about who controls the kitchen.” P10 [male, 38, hypertension] reported a similar pattern: “The products are well-designed and the videos are convincing. But I travel constantly for work. I can’t meal-prep when I’m in a hotel three days a week. The system assumes a stable home routine that I don’t have.” These accounts consistently point to environmental opportunity barriers—household dynamics, occupational constraints, residential instability—that override design-mediated motivation and capability. Critically, these participants did not lose trust or reduce their aesthetic appreciation; the failure occurred specifically at the COM-B opportunity component, suggesting that opportunity may function as a necessary gating condition that design features alone cannot address. Divergence pattern B: Social identity and community engagement as non-health adoption pathways. Three participants (P4, P8, P11) described engaging with the ecosystem primarily for social belonging, identity expression, or community membership rather than health improvement. This contrasts with the quantitative model’s assumption that health outcomes are the terminal goal of the cascade. P4 [female, 41, diabetes + hypertension] stated: “I keep buying the products partly because I feel like I belong to this community. When I post my meal photos in the group chat and everyone comments, that sense of belonging matters more to me than whether my blood sugar dropped. Using well-designed health products aligns with who I am—it says something about the kind of person I want to be.” P11 [female, 27, healthy] was explicit: “Honestly, I don’t have any health problems. I use these products because they’re aesthetically pleasing and my friends use them too. It’s a lifestyle statement. The health part is secondary.” These cases suggest an alternative pathway—design → identity alignment → social belonging → sustained engagement—that bypasses the health outcome reinforcement loop entirely. For these users, the “value” driving continued engagement is social and self-expression rather than health-functional, a distinction invisible to the quantitative model’s operationalization of perceived value. Divergence pattern C: Content credibility as the primary trust driver instead of design consistency. Five participants (P3, P5, P7, P9, P15) attributed their trust primarily to the substantive accuracy and professional quality of health content rather than to visual or design consistency. While the quantitative model positions design consistency as the primary upstream predictor of trust (β = 0.52), these accounts suggest that content credibility may be the deeper causal mechanism, with design consistency serving as a necessary but insufficient surface cue. P9 [female, 49, diabetes + hyperlipidemia] stated: “My HbA1c dropped from 7.8 to 6.9. That’s real results, not just nice-looking design. I trust this system because the nutritional advice is scientifically sound and it actually works—the design helped me stick with it, but the content is what convinced me.” P3 [female, 35, hypertension] concurred: “I’ve seen plenty of beautifully designed health apps that give terrible advice. The reason I trust this particular ecosystem is that the information matches what my doctor tells me. The consistent design is a bonus, but if the content were wrong, no amount of pretty colors would keep me.” These accounts raise the possibility of confounding between design quality and content quality—a limitation acknowledged in Section 4.5—and suggest that the large quantitative effect attributed to design consistency may partly reflect unmeasured content credibility.

### 3.9. Mixed-Method Integration: Convergence and Divergence

Qualitative findings served both convergent and divergent functions relative to the quantitative model, consistent with the analytic independence expected in rigorous mixed-method research. In terms of convergence, Themes 1–4 provided mechanistic depth for aggregate statistical patterns: visual–verbal consistency illuminated the psychological basis of the design–trust association (β = 0.52); aesthetic–usability synergy revealed how processing fluency and positive affect jointly generate emotional resonance (β = 0.71); tangible design features explained how COM-B capability and opportunity are scaffolded by product affordances; and outcome visibility clarified why health improvements strengthen value perceptions but not initial trust impressions. However, the independent analytic contribution of the qualitative data lies primarily in Theme 5, which identified three divergence patterns that challenge, qualify, or extend the quantitative model in ways that aggregate statistics cannot capture. First, divergence pattern A (high trust without adoption) reveals that the design → trust → intention → behavior cascade can stall at the intention–behavior junction when environmental opportunity barriers are present, even when all upstream psychological conditions are favorable. This finding challenges the quantitative model’s implicit assumption that the cascade is continuous: the qualitative evidence suggests a threshold model in which opportunity functions as a necessary gating condition rather than a graded mediator. The quantitative data corroborate this interpretation: among survey participants with above-median design consistency and trust scores (n = 68 in the longitudinal sample), those with below-median opportunity scores showed significantly lower mean adoption (M = 7.7, SD = 3.2) compared to the overall sample (M = 8.8, SD = 3.4), suggesting that opportunity deficits attenuate the design–adoption pathway even when trust is established. Second, divergence pattern B (social identity pathways) identifies a qualitatively distinct engagement mechanism that operates outside the health outcome reinforcement loop. For users motivated by social belonging and self-expression, the terminal “outcome” is identity congruence and community membership rather than biomarker improvement. This divergence has important theoretical implications: the cascade model implicitly treats health outcomes as the sole source of reinforcement, but the qualitative data suggest that social reinforcement may independently sustain engagement, particularly among younger and healthier participants. The quantitative model cannot distinguish these motivational pathways because both health-motivated and identity-motivated users may score similarly on behavioral adoption while pursuing fundamentally different goals. Third, divergence pattern C (content credibility as primary trust driver) raises a design–content confounding concern that qualifies the interpretation of the quantitative design consistency–trust association. While the SEM attributes a large direct effect to design consistency (β = 0.52), qualitative accounts suggest that perceived content accuracy may be the deeper trust mechanism, with design consistency serving as a surface-level cue that co-varies with content quality in this particular ecosystem. This divergence does not invalidate the quantitative finding but constrains its interpretation: the observed effect likely reflects a composite of design and content contributions that cannot be disentangled without experimental manipulation. Together, these convergences and divergences demonstrate the value of qualitative data as an independent analytic layer rather than merely an illustrative supplement. The divergent cases identify specific boundary conditions (opportunity as a gating mechanism), alternative causal pathways (identity-based engagement), and measurement confounds (design–content overlap) that are invisible to the aggregate quantitative model and that generate testable hypotheses for future research.

### 3.10. Sensitivity Analyses

To assess the robustness of our findings, we conducted several sensitivity analyses. First, we tested the structural model separately for participants with and without chronic conditions (diabetes, hypertension, or obesity). The pattern of significant paths remained consistent across both groups, with path coefficients differing by no more than 0.08 and all previously significant paths maintaining significance (all *p* < 0.05). This suggests that the design–behavior–health pathway operates similarly regardless of baseline health status.

Second, we examined potential gender differences through multi-group comparison. Although female participants showed slightly stronger design consistency → trust effects (β = 0.56 vs. β = 0.45 for males), the overall model fit was not significantly improved by allowing paths to vary by gender (Δχ^2^(14) = 18.3, *p* = 0.19), indicating gender invariance.

Third, we tested alternative model specifications, including a direct path from design consistency to purchase intention (bypassing psychological mediators) and a direct path from aesthetic experience to behavioral adoption. Neither alternative specification improved model fit or explained additional variance, supporting our theorized mediation pathways.

Finally, we conducted bootstrapped analysis with 20,000 resamples (double the original 10,000) to verify the stability of indirect effects. All previously reported indirect effects remained significant with nearly identical point estimates and confidence intervals, confirming the robustness of our mediation findings.

## 4. Discussion

### 4.1. Summary of Findings

This study tested 17 hypotheses organized into six conceptual groups, achieving strong empirical support (Table 3). All hypotheses in Groups 1–5 (H1–H5, 15 hypotheses) were fully supported, demonstrating robust evidence for the proposed cascade model linking design features to health outcomes.

Stage 1 (Design Effects): Design consistency significantly influenced all three psychological mediators (H1a–c supported), and aesthetic experience both directly predicted emotional resonance and amplified consistency effects through moderation (H2a–b supported). This dual pathway—direct effects and moderation—suggests that design consistency and aesthetic experience may function synergistically rather than independently.

Stage 2 (Psychological → Behavioral Intention): Trust, perceived value, and reduced risk jointly predicted purchase intention as hypothesized (H3a–c supported), with perceived value showing the strongest effect (β = 0.51). The substantial variance (R^2^ = 0.64) indicates that these three perceptual mechanisms capture much of the psychological basis for behavioral intentions in digital health contexts.

Stage 3 (Intention → Behavior): The COM-B framework successfully bridged the intention–behavior gap, with all three components mediating the relationship and collectively explaining 75% of the total effect (H4a–c supported). This finding addresses a persistent challenge in health behavior research: the association between intention and actual behavior. The partial mediation (significant direct effect remained) suggests that while COM-B mechanisms are important correlates of adoption, other unmeasured factors also contribute to adoption.

Stage 4 (Behavior → Health): High behavioral adoption was associated with clinically meaningful improvements across all four health indicators (H5a-d supported), with effect sizes ranging from d = 0.51 to d = 0.89, representing medium-to-large effects by Cohen’s standards. Notably, the weight loss achieved by high-adoption users (2.8 kg) represents 5% body weight loss for a person weighing 56 kg, meeting clinical thresholds for metabolic benefit. Similarly, the 0.7% HbA1c reduction exceeds the 0.5% threshold which is considered clinically significant for diabetes management. It is essential to interpret these associations with conceptual care. The behavioral adoption composite index used in this study encompasses commercial engagement (purchase frequency) alongside behavioral proxies (usage consistency, engagement depth); it is not a direct measure of health behavior change. The observed co-occurrence of higher adoption scores with improved biomarkers is plausibly explained by a convergence of mechanisms: consistent product use may have increased dietary compliance (e.g., structured meal-replacement adherence), while frequent platform engagement may have reinforced health-relevant behavioral routines. Nevertheless, because adoption and biomarker outcomes were not experimentally linked, and because concurrent lifestyle changes were not systematically controlled, the observed associations between adoption level and clinical improvement should not be interpreted as evidence of a direct effect of commercial product purchasing per se on metabolic improvement. Future research employing validated, condition-specific health behavior instruments alongside commercial engagement metrics will be needed to disentangle these dimensions.

Stage 5 (Feedback Loop): Health outcomes were positively associated with repurchase intention as hypothesized (H6a supported), with users achieving clinically meaningful improvements, showing 52% higher odds of continued engagement. However, H6b received only partial support: while health improvements strengthened the perceived value pathway (β = 0.24, *p* < 0.05), they did not significantly reinforce the direct design consistency → trust relationship (β = 0.11, ns). This suggests that the feedback mechanism operates primarily through value perceptions and outcome satisfaction rather than retrospectively altering initial design impressions.

The high hypothesis confirmation rate (94% full support, 6% partial support) provides strong validation for the integrated cascade model and is consistent with robust associations between design features, psychological processes, behavioral adoption, and objective health outcomes. The single partially supported hypothesis (H6b) offers important theoretical insight into the temporal dynamics of design perceptions and warrants further investigation. Importantly, these findings are associational in nature, grounded in a single-ecosystem observational design, and should be interpreted accordingly. Ecosystem-specific characteristics, including the influencer’s established credibility, the homogeneous socioeconomic profile of participants, and the cultural context of Chinese digital health commerce, may have amplified the observed associations. Several potential confounding factors merit explicit consideration. The influencer’s personal credibility, communication style, and established reputation likely contributed to the observed trust and engagement effects, independent of design consistency per se. Users’ pre-existing health motivation and self-selection into the ecosystem may have inflated associations between adoption and health outcomes. Concurrent lifestyle changes—including dietary modifications, exercise routines, or medication adjustments—were not systematically controlled, making it impossible to attribute observed biomarker improvements solely to ecosystem engagement. Additionally, the co-variation in design quality with content quality in this ecosystem means that the independent effect of design cannot be isolated without experimental manipulation. Readers are, therefore, cautioned against generalizing these results to other digital health platforms, population groups, or cultural settings without further empirical validation.

### 4.2. Theoretical Mechanisms and Contributions

#### 4.2.1. Design Consistency as a Multi-Layered Credibility Heuristic

Our finding that design consistency was strongly associated with trust (β = 0.52, *p* < 0.001) extends dual-process persuasion theories [38] to visual design in health contexts, revealing three complementary mechanisms that operate synergistically. The first mechanism is cognitive fluency: Visual consistency reduces processing effort, creating subjective ease that users misattribute to source credibility. This fluency-based interpretation finds support in our mediation finding (aesthetic experience → emotional resonance, β = 0.71, *p* < 0.001), demonstrating that visually coherent designs facilitate smoother cognitive processing [39,40], which in turn is associated with positive affective responses. The Aesthetic–Usability Effect literature demonstrates that processing fluency from visual harmony has been linked to perceived trustworthiness even when objective credibility indicators are controlled, suggesting that users may employ aesthetic coherence as a cognitive shortcut for reliability judgments.

The second mechanism is competence signaling: Maintaining cross-platform design consistency requires substantial organizational coordination, resource allocation, and quality control systems—investments that function as costly signals [41,42] that only capable providers can sustain. Users intuitively detect this signal, reasoning that organizations demonstrating discipline in visual presentation likely maintain equivalent rigor in product development and safety protocols. This costly signaling mechanism, originally developed to explain biological displays and economic credentials, applies powerfully to digital health contexts where users must assess provider competence without direct verification opportunities. The magnitude of our trust coefficient (β = 0.52) notably exceeds typical brand trust effects in non-health e-commerce (β ≈ 0.30) [43,44], suggesting domain-specific amplification where health stakes elevate the importance of credibility assessment, and users attend more carefully to consistency cues.

The third mechanism is schema reinforcement: Design consistency strengthens brand schema formation [45], reducing uncertainty in unfamiliar health product contexts where users lack expertise to evaluate substantive claims. Repeated exposure to consistent visual elements across platforms facilitates schema consolidation, creating stable mental representations that serve as familiarity-based trust anchors. This mechanism operates particularly powerfully for health influencers navigating the transition from content creator to product vendor—a novel role configuration where traditional credibility markers (medical credentials, institutional affiliations) may be absent but visual professionalism can substitute as a legitimacy signal.

The convergence of these three mechanisms—fluency, signaling, and schema reinforcement—explains why our design consistency effect substantially exceeds previous estimates from commercial contexts. In health domains characterized by high consequences, information asymmetry, and vulnerability, users amplify their reliance on peripheral cues when central route processing is constrained by limited expertise. Design consistency, as a readily observable and intuitively interpretable cue, thus assumes outsized importance in shaping initial trust formation.

#### 4.2.2. COM-B as Action Control: Bridging the Intention–Behavior Gap

Our finding that COM-B components collectively mediate 75% of the purchase intention → behavioral adoption pathway (total indirect effect = 0.51, *p* < 0.001) addresses a persistent challenge in health psychology: the notorious intention–behavior gap. Meta-analytic evidence indicates that intentions explain only 20–30% of variance in health behaviors [46,47,48], leaving the majority of behavioral variance unaccounted for by motivational constructs alone. Our results suggest that decomposing behavioral adoption through the COM-B lens—capability (knowledge, skills), opportunity (environmental affordances), and motivation (sustained impetus)—is associated with a substantially narrower gap, with unexplained variance reduced to approximately 25%.

This finding extends action control frameworks by identifying the mechanisms associated with the pathway from favorable intentions to enacted behaviors. Capability encompasses the knowledge and skills associated with behavioral self-regulation; in our context, users reported needing nutritional literacy and meal planning competencies to implement dietary recommendations. Opportunity encompasses environmental scaffolding associated with reduced self-control demands; the platform provided meal delivery infrastructure and social accountability features that externalized behavioral support. Motivation captures sustained commitment despite obstacles and temptations; emotional resonance with health content was associated with maintained volitional strength during challenging implementation phases.

Notably, the nearly equivalent effect sizes of capability (β = 0.31, *p* < 0.01), opportunity (β = 0.28, *p* < 0.01), and motivation (β = 0.34, *p* < 0.001) challenge motivation-centric assumptions prevalent in health behavior theories. Rather than motivation serving as the primary driver with capability and opportunity as enablers, our structural model reveals that all three components function as co-equal necessary conditions—individually insufficient but collectively sufficient for behavioral translation. This tripartite sufficiency pattern suggests that interventions targeting only one component may face ceiling effects, whereas integrated approaches addressing all three dimensions may yield multiplicative rather than additive gains.

The partial mediation finding (direct effect β = 0.17, *p* < 0.05 remains significant) indicates that COM-B accounts for approximately three-quarters of the intention–behavior translation, with the remaining quarter potentially attributable to habit formation, social norms, or environmental cues not captured in our model. Future research should investigate whether automated behavioral patterns, once established through initial COM-B facilitation, begin to operate independently of ongoing capability, opportunity, and motivation fluctuations.

#### 4.2.3. Health Outcomes as Reinforcement: The Feedback Loop Mechanism

Our finding that objective health improvements are associated with sustained engagement (OR = 1.52, 95% CI [1.21, 1.91], *p* < 0.01) represents a novel theoretical contribution often overlooked in health behavior models: the bidirectional relationship between outcomes and determinants. Traditional frameworks treat behavior as the terminal endpoint, rarely examining how behavioral consequences feed back to shape future motivation, capability perceptions, or opportunity recognition. Real-world health behavior, however, unfolds dynamically across time, with outcome experiences recursively updating the psychological determinants that originally facilitated action.

The temporal asymmetry revealed in our supplementary moderation analyses provides crucial mechanistic insight: health improvements showed stronger associations with perceived value (β = 0.24, *p* < 0.05) than on trust (β = 0.11, ns). This pattern suggests differential susceptibility to experiential revision. Trust, formed primarily through design consistency cues during initial exposure, exhibits stability and resistance to updating—once established through peripheral route processing, initial trust judgments anchor subsequent evaluations. In contrast, perceived value showed dynamic updating based on accumulated outcome evidence, with users continuously recalibrating value assessments as health benefits accrued or failed to materialize.

This finding aligns with self-perception theory [49,50]: individuals infer their attitudes by observing their own behaviors and outcomes, reasoning backward from tangible results to retrospective value judgments. Users experiencing meaningful health improvements may think, “I continue using this product and my health is improving; therefore, this product must be valuable”—an attribution that retroactively justifies initial adoption and motivates continued engagement. This self-perception mechanism may operate most powerfully when outcomes are salient, personally relevant, and clearly attributable to the focal behavior rather than confounded by external factors.

We propose a Two-Phase Engagement Model as a novel theoretical contribution: Phase 1 (acquisition) relies heavily on design consistency → trust pathways, where peripheral cues establish initial credibility in the absence of direct experience. Phase 2 (retention) increasingly depends on health outcomes → value perceptions, where accumulated experiential evidence supersedes initial impressions. This temporal model reconciles apparent contradictions in the health technology adoption literature, where design quality predicts trial but not sustained use, and outcome effectiveness predicts retention but not trial. Optimal engagement strategies must, therefore, employ phase-appropriate tactics: invest in design excellence to maximize acquisition and deliver measurable health improvements to ensure retention.

The practical implication is sobering: even exceptional design consistency may not compensate for products that fail to deliver objective health benefits. Users may initially trust and purchase based on aesthetic coherence, but sustained engagement likely requires experientially verifiable value. Digital health interventions must therefore prioritize clinical effectiveness alongside interface design, recognizing that these elements address distinct phases of the engagement lifecycle and neither alone suffices for long-term success.

### 4.3. Practical Implications

Our findings extend health communication theory in three ways. First, results suggest that design consistency may function through dual pathways: as a heuristic cue signaling professionalism (peripheral route) and by reducing cognitive load to facilitate systematic processing (central route) in the Elaboration Likelihood Model [51]. This integration challenges the traditional separation of design aesthetics, consumer psychology, and health behavior change into isolated domains.

Second, we integrate the COM-B framework with consumer psychology, suggesting that product design may need to address all three COM-B components for purchase intentions to be associated with sustained behavioral adoption. This integration contributes to understanding the intention–behavior gap that plagues digital health interventions [41,52]. The finding that COM-B components were associated with 75% of the intention–adoption relationship supports the framework’s potential utility beyond traditional behavioral interventions.

Third, we identify an associational feedback loop whereby health outcomes were positively associated with repurchase intention, consistent with self-perception processes [46,47], contributing to understanding long-term health behavior maintenance. The partial support for H6b reveals important temporal dynamics: initial design impressions may form rapidly and persist once established, making them less susceptible to retrospective revision even when outcomes validate those impressions. This suggests that design consistency’s primary impact occurs during initial engagement rather than through outcome-driven retrospection.

Mechanism of design consistency effects: As elaborated in Section 4.2.1, the design consistency–trust association (β = 0.52, *p* < 0.001) substantially exceeds typical design effects in consumer research (β ≈ 0.20–0.35) [53]. Three complementary mechanisms—cognitive fluency [39,40], competence signaling [41,42], and identity alignment [52]—likely operate synergistically to account for this amplified effect in health contexts where credibility assessment is paramount.

Bridging the intention–behavior gap through COM-B: As detailed in Section 4.2.2, COM-B components collectively mediated 75% of the intention–adoption association (total indirect effect = 0.51, *p* < 0.001), substantially exceeding the 20–30% of behavioral variance typically explained by intentions alone [41,52], leaving a substantial “gap” unexplained. The partial mediation pattern (direct effect β = 0.17, *p* < 0.05) suggests that additional unmeasured factors (habit formation, social norms, environmental cues) contribute to the remaining quarter. This finding is consistent with theoretical developments by Sheeran and Webb [54] who argue that action control mechanisms may moderate the intention–behavior relationship, with the nearly equivalent COM-B effect sizes (β = 0.31, 0.28, 0.34) challenging the assumption that motivation alone is primary.

Novel contribution: The positive reinforcement loop from outcomes to engagement. As elaborated in Section 4.2.3, the association between objective health improvements and continued engagement (OR = 1.52, 95% CI [1.21, 1.91]) supports a Two-Phase Engagement Model: Phase 1 (acquisition) relies on design consistency–trust pathways, while Phase 2 (retention) depends on health outcomes–value perceptions. Traditional health behavior models rarely examine such feedback loops [50]. The temporal asymmetry between stable trust impressions and dynamic value updating, consistent with self-perception theory [46], has practical implications: design excellence may facilitate acquisition, but sustained engagement likely requires delivering measurable health results that users can experientially verify.

### 4.4. Theoretical Implications

For digital health product developers, our findings offer actionable principles. First, invest in rigorous cross-platform design consistency across all touchpoints, as this is associated with enhanced trust and value perceptions. While the current evidence is observational, the magnitude and consistency of associations across measurement models suggests this is a practically important domain for intervention design. Establish comprehensive brand guidelines covering visual identity, terminology, and interaction patterns, and rigorously enforce them. Our results show that consistency effects are substantial (β = 0.52 for trust, β = 0.68 for value), suggesting that design consistency is not merely cosmetic but may function as an important correlate of user engagement in digital health contexts.

Second, prioritize comprehensive COM-B targeting: Educational content building capability, convenient access and portion control creating opportunity, and tracking mechanisms enhancing motivation are equally important. Our mediation analysis shows that motivation alone (β = 0.34) cannot compensate for deficits in capability (β = 0.31) and opportunity (β = 0.28). Digital health interventions that focus solely on motivation risk underperformance.

Third, integrate objective outcome tracking and make improvements visible to users, as tangible results were associated with powerful reinforcement loops in this study (OR = 1.52 for continued engagement). The feedback effect operates primarily through perceived value rather than trust, suggesting that outcome visibility should emphasize concrete benefits and value delivery.

Fourth, recognize that aesthetic quality appears to signal competence and professionalism in health contexts where trust is paramount. The moderation effect (Δχ^2^ = 8.4) indicates that aesthetic experience was associated with amplified consistency effects by 68% (high aesthetics β = 0.64 vs. low aesthetics β = 0.38). This is not mere beautification but a critical credibility mechanism.

### 4.5. Limitations and Future Research

Several limitations warrant consideration. First, the observational, non-randomized design is a fundamental constraint: all quantitative and qualitative data derive from a single digital health ecosystem, and exposure to design consistency was neither experimentally manipulated nor controlled. Accordingly, all findings must be interpreted as associational rather than causal. Observed associations between design features and health outcomes may reflect confounding with unmeasured factors, including the influencer’s personal credibility, prior brand loyalty among followers, and cultural dispositions toward health consumerism in the specific context of Chinese digital commerce. Future randomized controlled trials manipulating specific design features across experimental and control conditions are required to establish causal pathways.

Second, the single-ecosystem case study severely limits external validity. All participants were self-selected users embedded within one influencer-centered commercial ecosystem, raising questions about whether findings generalize to digital health platforms with different governance structures, professional health providers, or culturally distinct user bases. Ecosystem-specific factors, including the influencer’s credibility, the platform’s cultural embeddedness in Chinese digital commerce, and the homogeneous socioeconomic profile of the sample (78% with bachelor’s degree or higher; 42% with household income above 150K RMB annually), may have amplified the observed effects. Claims regarding broader digital health ecosystems should be understood as exploratory and bounded to this single-case context pending multi-site replication.

Third, common method variance is a concern given that most constructs (design consistency, aesthetic experience, trust, perceived value, perceived risk, emotional resonance, purchase intention, and COM-B components) were measured via self-report within the same survey instrument. Although the inclusion of objective health biomarkers (HbA1c, LDL-C, fasting glucose, weight) substantially mitigates this concern for the downstream health outcome analyses, self-report data for the psychological and behavioral constructs may be susceptible to shared-method bias. We conducted Harman’s single-factor test as a diagnostic check: the first unrotated factor explained 34.2% of total variance (well below the 50% threshold). Additionally, we conducted a CFA-based common latent factor (CLF) analysis by adding a single unmeasured latent factor to the measurement model and allowing it to load on all observed indicators. The method factor accounted for approximately 18% of shared variance, below the 25% threshold typically regarded as problematic. Furthermore, comparison of standardized regression weights between the baseline CFA model and the CLF-adjusted model revealed no substantial differences (all Δβ < 0.05), suggesting that common method variance, while present, does not substantively alter the pattern of structural relationships. Nonetheless, these post hoc statistical remedies cannot fully eliminate CMV concerns. Procedural design elements, including temporally separated measurement of behavioral adoption and health outcomes and use of different response formats across construct families, further reduce this risk. Nevertheless, readers should interpret self-report associations with appropriate caution.

Fourth, the behavioral adoption composite index conflates commercial engagement (purchase frequency) with health behavior change (usage consistency, engagement depth). While this operationalization serves as a practical proxy, it may not fully capture meaningful lifestyle modification independent of product purchasing. Future research should employ validated, condition-specific health behavior measures alongside commercial engagement metrics to disentangle these dimensions. Fifth, the 3-month follow-up period, while adequate for detecting initial metabolic changes, does not address long-term maintenance. Future studies extending follow-up to 12 months or beyond are needed to determine whether design-associated improvements are sustained. Additionally, respondent burden and fatigue effects, as well as social desirability bias in self-report measures, represent potential sources of measurement error that warrant attention in future research. Sixth, the distinction between design quality and content quality is not fully disentangled in the current analysis. Although the study attributes effects to design consistency, qualitative findings suggest that content credibility also plays a key role in shaping trust. Because design quality and content quality co-vary in this ecosystem, a well-designed platform is also likely to feature carefully curated content—the independent contribution of design features cannot be isolated from content effects without experimental manipulation. Future research should employ factorial designs that independently vary design consistency and content quality to clarify their relative and interactive contributions to trust and behavioral adoption. Seventh, health literacy and socioeconomic factors, including digital device access, income, and prior health knowledge, likely moderate the associations observed here but were not systematically examined. Users with low health literacy or limited financial resources may have systematically lowered engagement with and benefitted from digital health ecosystems of this type. Future research should examine boundary conditions of design effects across populations varying in health literacy, digital fluency, age, and socioeconomic background.

The partial support for H6b warrants further discussion. We hypothesized that health improvements would retrospectively strengthen users’ perceptions of design consistency’s role in building trust. While outcomes did reinforce perceived value (supporting the feedback loop), they did not significantly moderate the design consistency → trust relationship. This may reflect temporal ordering: initial design impressions form rapidly and persist once established, making them less susceptible to retrospective revision even when outcomes validate those impressions. Alternatively, users may attribute health improvements to their own effort or the intervention content rather than to design consistency per se. Future research using longitudinal within-person designs could better capture how outcome experiences shape evolving design perceptions over time.

Future research should pursue several complementary directions. First, randomized controlled trials that experimentally manipulate specific design features (e.g., consistency vs. inconsistency across platforms) are needed to establish causal mechanisms and isolate design effects from content quality and influencer credibility. Second, multi-site replication across culturally diverse ecosystems, including platforms governed by professional healthcare providers in Western, lower-income, and health-literacy-diverse contexts, is essential for assessing external validity. Third, studies examining moderating roles of health literacy, socioeconomic status, age, sex, and digital fluency would help identify for whom design effects are strongest and clarify boundary conditions. Fourth, extending follow-up periods to 12 months or beyond is needed to determine whether design-associated health improvements are sustained over time. Fifth, future work should employ validated, condition-specific health behavior measures to disentangle commercial engagement from meaningful lifestyle modification. Sixth, investigating the integrative role of artificial intelligence, including AI-driven personalization of design elements, real-time adaptive interfaces, and AI-mediated health coaching, represents a promising frontier for next-generation digital health ecosystem design. Finally, the relative contributions of economic factors (product price, socioeconomic access), social influences (impression management, peer norms), and psychological mechanisms (as examined here) to digital health adoption warrant comparative investigation.

## 5. Conclusions

This study provides evidence that design consistency and aesthetic experience were significantly associated with key indicators of digital health effectiveness. The results show that design is associated with trust, perceived value, and emotional resonance, which in turn co-occur with behavioral adoption through COM-B mechanisms. When sustained, these behaviors were associated with measurable health improvements that reinforced engagement, consistent with possible positive feedback cycles. The high hypothesis support rate (94%) is consistent with the integrated cascade model and provides preliminary, context-specific design principles that may inform digital health product development within similar influencer-centered ecosystems, though generalizability to other contexts requires empirical validation. As digital health ecosystems proliferate, rigorous empirical research linking design to health outcomes becomes essential for evidence-based practice.

## Figures and Tables

**Table 1:** Sample demographic characteristics (N = 186).

Characteristic	n	% or M (SD)
Gender		
Female	141	75.8%
Male	45	24.2%
Age (years)	186	45.2 (13.1)
Range		22–68
Education		
Bachelor’s degree or higher	145	78.0%
Below bachelor’s degree	41	22.0%
Household Income		
>150K RMB annually	78	42.0%
≤150K RMB annually	108	58.0%
Chronic Conditions *		
Type 2 Diabetes	52	28.0%
Hypertension	71	38.2%
Dyslipidemia	48	25.8%
Obesity (BMI ≥ 28)	45	24.2%
Platform Engagement		
Usage duration (months)	186	8.7 (4.2)
Videos viewed	186	47.3 (28.6)
Products purchased	186	3.8 (2.1)

Note: * Chronic conditions are not mutually exclusive. M = Mean; SD = Standard Deviation.

**Table 2:** Descriptive statistics, reliabilities, and correlations.

Variable	M	SD	1	2	3	4	5	6	7
1. Design Consistency	5.82	0.94	(0.89)						
2. Aesthetic Experience	5.47	1.02	0.68 ***	(0.91)					
3. Trust	5.63	0.97	0.52 ***	0.59 ***	(0.92)				
4. Perceived Value	5.51	0.89	0.68 ***	0.64 ***	0.71 ***	(0.90)			
5. Perceived Risk (reversed)	5.28	1.14	−0.41 ***	−0.38 ***	−0.56 ***	−0.49 ***	(0.87)		
6. Emotional Resonance	5.39	1.07	0.55 ***	0.71 ***	0.66 ***	0.69 ***	−0.43 ***	(0.88)	
7. Purchase Intention	5.44	1.11	0.48 ***	0.51 ***	0.63 ***	0.72 ***	−0.46 ***	0.64 ***	(0.91)

Note: N = 186. Diagonal values in parentheses (bolded) are Cronbach’s α reliability coefficients. All scales used 7-point Likert response format (1 = strongly disagree, 7 = strongly agree). *** *p* < 0.001.

**Table 3:** Measurement model validation result.

Construct	Items	Factor Loading Range	α	CR	AVE	√AVE
Design Consistency	6	0.71–0.84	0.89	0.90	0.60	0.77
Aesthetic Experience	8	0.74–0.88	0.91	0.92	0.61	0.78
Trust	6	0.78–0.91	0.92	0.92	0.66	0.81
Perceived Value	8	0.68–0.86	0.90	0.91	0.58	0.76
Perceived Risk	6	0.64–0.82	0.87	0.88	0.55	0.74
Emotional Resonance	5	0.72–0.87	0.88	0.89	0.62	0.79
Purchase Intention	4	0.81–0.89	0.91	0.91	0.72	0.85
COM-B Capability	4	0.70–0.84	0.86	0.87	0.62	0.79
COM-B Opportunity	6	0.66–0.81	0.84	0.86	0.54	0.73
COM-B Motivation	7	0.73–0.88	0.89	0.90	0.58	0.76

Note: N = 186. α = Cronbach’s alpha; CR = composite reliability; AVE = average variance extracted; √AVE = square root of AVE (bolded in last column). Discriminant validity established: √AVE for each construct exceeded inter-construct correlations (see Table 2). Confirmatory factor analysis model fit: χ^2^(847) = 1243.2, *p* < 0.001 (expected given sample size), CFI = 0.94, TLI = 0.93, RMSEA = 0.05 [90% CI: 0.04, 0.06], SRMR = 0.06. All fit indices exceeded recommended thresholds.

**Table 4:** Structural path coefficients.

Path	Hypothesis	β	SE	t	*p*	95% CI	R^2^
Design Consistency → Trust	H1a	0.52	0.06	8.67	<0.001	[0.40, 0.64]	0.27
Design Consistency → Perceived Value	H1b	0.68	0.05	13.60	<0.001	[0.58, 0.78]	0.46
Design Consistency → Perceived Risk	H1c	−0.41	0.07	−5.86	<0.001	[−0.55, −0.27]	0.17
Aesthetic Experience → Emotional Resonance	H2a	0.71	0.04	17.75	<0.001	[0.63, 0.79]	0.50
Trust → Purchase Intention	H3a	0.28	0.07	4.00	<0.001	[0.14, 0.42]	—
Perceived Value → Purchase Intention	H3b	0.51	0.06	8.50	<0.001	[0.39, 0.63]	—
Perceived Risk → Purchase Intention	H3c	−0.19	0.06	−3.17	0.002	[−0.31, −0.07]	0.64
Purchase Intention → Capability	H4a	0.54	0.06	9.00	<0.001	[0.42, 0.66]	0.29
Purchase Intention → Opportunity	H4b	0.48	0.07	6.86	<0.001	[0.34, 0.62]	0.23
Purchase Intention → Motivation	H4c	0.61	0.05	12.20	<0.001	[0.51, 0.71]	0.37
Capability → Behavioral Adoption	H4a	0.31	0.09	3.44	0.001	[0.13, 0.49]	—
Opportunity → Behavioral Adoption	H4b	0.28	0.09	3.11	0.002	[0.10, 0.46]	—
Motivation → Behavioral Adoption	H4c	0.34	0.08	4.25	<0.001	[0.18, 0.50]	0.62
Purchase Intention → Behavioral Adoption (direct)	—	0.17	0.08	2.13	0.033	[0.01, 0.33]	—

Note: N = 186. β = standardized path coefficient; SE = standard error; t = t-value; CI = confidence interval. R^2^ values (bolded in last column) shown for endogenous variables. Structural model fit: χ^2^(247) = 358.4, *p* < 0.001, CFI = 0.95, TLI = 0.94, RMSEA = 0.05 [90% CI: 0.04, 0.06], SRMR = 0.06. All confidence intervals are bias-corrected bootstrap estimates (10,000 iterations).

**Table 5:** Moderation analysis details.

Aesthetic Experience Level	β (DC → Trust)	SE	t	*p*	95% CI	Effect Size
Low Level (−1 SD; M = 4.45)	0.38	0.09	4.22	0.008	[0.20, 0.56]	Medium
Mean Level (M = 5.47)	0.52	0.06	8.67	<0.001	[0.40, 0.64]	Large
High Level (+1 SD; M = 6.49)	0.64	0.08	8.00	<0.001	[0.48, 0.80]	Large
Difference (High - Low)	0.26	0.12	2.17	0.030	[0.02, 0.50]	68% increase

Note: N = 186. DC = design consistency. Multi-group comparison confirmed significant moderation: Δχ^2^(1) = 8.4, *p* < 0.01. Simple slopes analysis shows aesthetic experience amplifies design consistency effects by 68% when comparing high versus low aesthetic experience groups. The Johnson–Neyman technique identified the significance region: design consistency → trust effects became significant when aesthetic experience exceeded 3.2 on the 1–7 scale. Effect size interpretation: Small < 0.20, Medium 0.20–0.50, Large > 0.50.

**Table 6:** Clinical significance of health outcomes by behavioral adoption level.

Outcome and Clinical Threshold	High Adoption n (%)	Medium Adoption n (%)	Low Adoption n (%)	χ^2^(2)	*p*	NNT	Effect Size
Weight Loss ≥ 5%	19 (53%)	14 (20%)	2 (6%)	24.6	<0.001	2.1	Large
Fasting Glucose ≥ 0.5 mmol/L	22 (61%)	24 (34%)	5 (14%)	18.9	<0.001	2.1	Large
HbA1c Reduction ≥ 0.5%	24 (67%)	22 (31%)	4 (11%)	27.8	<0.001	1.8	Large
LDL-C Reduction ≥ 0.3 mmol/L	18 (50%)	21 (30%)	6 (17%)	10.7	0.005	3.0	Medium
Any Clinically Meaningful Change	31 (86%)	40 (56%)	10 (28%)	28.4	<0.001	1.7	Large

Note: N = 143 with complete 3-month follow-up data. High adoption: n = 36 (behavioral adoption score 12–18); medium adoption: n = 71 (score 6–11); low adoption: n = 36 (score 0–5). NNT = number needed to treat, calculated as the number of participants who would need to move from low to high adoption level for one additional person to achieve the clinical threshold. “Any clinically meaningful change” = achieving threshold on at least one of the four health indicators. Effect size based on Cramér’s V: Small < 0.10, Medium 0.10–0.30, Large > 0.30. Clinical thresholds based on established guidelines for meaningful health improvements in lifestyle medicine interventions.

**Table 7:** Summary of hypothesis testing results.

Hypothesis	Prediction	Result
H1a	Design consistency → Trust (+)	β = 0.52 ***
H1b	Design consistency → Perceived value (+)	β = 0.68 ***
H1c	Design consistency → Perceived risk (−)	β = −0.41 ***
H2a	Aesthetic experience → Emotional resonance (+)	β = 0.71 ***
H2b	Aesthetic experience moderates consistency → trust	Δχ^2^(1) = 8.4 **
H3a	Trust → Purchase intention (+)	β = 0.28 ***
H3b	Perceived value → Purchase intention (+)	β = 0.51 ***
H3c	Perceived risk → Purchase intention (−)	β = −0.19 **
H4a	Capability mediates intention → adoption	Indirect = 0.17 **
H4b	Opportunity mediates intention → adoption	Indirect = 0.13 **
H4c	Motivation mediates intention → adoption	Indirect = 0.21 ***
H5a	Adoption → Weight improvement	d = 0.89 ***
H5b	Adoption → Glucose improvement	d = 0.72 ***
H5c	Adoption → HbA1c improvement	d = 0.65 ***
H5d	Adoption → LDL-C improvement	d = 0.51 **
H6a	Health outcomes → Repurchase intention (+)	OR = 1.52 ***
H6b	Outcomes strengthen design–trust relationship	β = 0.11 (ns)

Note: *** *p* < 0.001, ** *p* < 0.01. H6b was partially supported: health outcomes strengthened perceived value → repurchase (β = 0.24, *p* < 0.05) but not design consistency → trust (β = 0.11, ns). Overall confirmation rate: 94% full support, 6% partial support.

**Table 8:** Themes and sub-themes identified from thematic analysis.

Theme	Sub-Themes
Theme 1: Visual–verbal consistency as credibility marker	Design coherence signals professionalism Consistency functions as expertise heuristic Multi-platform coherence builds trust
Theme 2: Aesthetic–usability synergy	Beauty and function perceived as integrated Visual appeal reduces cognitive friction Aesthetic quality signals reliable functionality
Theme 3: Tangible design enabling self-regulation	Portion control features reduce decision burden Physical design supports self-regulatory capacity Product features automate healthy choices
Theme 4: Outcome visibility reinforcing commitment	Health improvements validate ecosystem value Users attribute success to personal effort Outcomes strengthen engagement through self-perception
Theme 5: Divergent cases—boundary conditions of the design–adoption pathway	A: High trust/design without adoption (opportunity barriers) B: Social identity and community as non-health pathways C: Content credibility as primary trust driver, not design

Note: N = 15 interview participants. Themes emerged from systematic thematic analysis following Braun and Clarke’s (2006) [35] six-phase framework. Inter-coder reliability: κ = 0.81 (substantial agreement). Saturation achieved after 14 interviews. Themes 1–4 converge with quantitative pathways by revealing underlying psychological mechanisms. Theme 5 represents an independent qualitative analytic contribution, identifying divergences and boundary conditions that challenge or qualify the aggregate quantitative model. Each theme is illustrated with representative quotes in the main text.

## Data Availability

The data that support the findings of this study are available from the corresponding author upon reasonable request, subject to privacy and ethical restrictions.

## Abbreviations

The following abbreviations are used in this manuscript:

COM-B	Capability, Opportunity, Motivation Behavior model
SEM	Structural Equation Modeling
CFI	Comparative Fit Index
TLI	Tucker–Lewis Index
RMSEA	Root Mean Square Error of Approximation
SRMR	Standardized Root Mean Square Residual
AVE	Average Variance Extracted
CR	Composite Reliability
HbA1c	Glycated Hemoglobin
LDL-C	Low-Density Lipoprotein Cholesterol
BMI	Body Mass Index
OR	Odds Ratio
NNT	Number Needed to Treat
